# Seasonal Influenza Vaccine Effectiveness in Preventing Laboratory Confirmed Influenza in 2014-2015 Season in Turkey: A Test-Negative Case Control Study

**DOI:** 10.4274/balkanmedj.2017.0487

**Published:** 2018-01-20

**Authors:** Can Hüseyin Hekimoğlu, Mestan Emek, Emine Avcı, Selmur Topal, Mustafa Demiröz, Gül Ergör

**Affiliations:** 1 Department of Microbiology Reference Laboratories, Public Health Institute of Turkey, Ankara, Turkey; 2 Antalya Public Health Directorate, Antalya, Turkey; 3 Department of Infectious Diseases, Public Health Institute of Turkey, Ankara, Turkey; 4 Bursa Public Health Directorate, Bursa, Turkey; 5 Department of Public Health, Dokuz Eylül University School of Medicine, İzmir, Turkey

**Keywords:** Influenza, seasonal, vaccine, surveillance, effectiveness

## Abstract

**Background:**

Influenza has an important public health impact worldwide with its considerable annual morbidity among persons with or without risk factors and its serious complications among persons in high-risk groups. The seasonal influenza vaccine is essential for preventing the burden of influenza in a population. Since the vaccine is reformulated each season according to the virus serotypes in circulation, its effectiveness can vary from season to season. Vaccine effectiveness is defined as the relative risk reduction in vaccinated individuals in observational studies.

**Aims:**

To calculate influenza vaccine effectiveness in preventing laboratory-confirmed influenza in the Turkish population for the first time using the national sentinel surveillance data in the 2014-2015 influenza season.

**Study Design:**

Test-negative case-control study.

**Methods:**

We compared vaccination odds of influenza positive cases to influenza negative controls in the national influenza surveillance in Turkey to estimate influenza vaccine effectiveness.

**Results:**

The influenza vaccine effectiveness against influenza A (H1N1) (68.4%, 95% CI: -2.9 to 90.3) and B (44.6%, 95% CI: -27.9 to 66.6) were moderate, and the influenza vaccine effectiveness against influenza A (H3N2) (75.0%, 95% CI: -86.1 to 96.7) was relatively high; all had low precision given the low vaccination coverage. Overall, the influenza vaccination coverage rate was 4.2% (95% CI: 3.5 to 5.0), which is not sufficient to control the burden of influenza.

**Conclusion:**

In Turkey, national surveillance for influenza should be strengthened and utilised annually for the assessment of influenza vaccine effectiveness with more precision. Annual influenza vaccine effectiveness in Turkey should continue to be monitored as part of the national sentinel influenza surveillance.

Influenza has an important public health impact worldwide with its considerable annual morbidity among persons with or without risk factors and its serious complications among persons in high-risk groups. Seasonal influenza vaccine is essential for preventing the burden of influenza (incidence, hospitalisation, mortality and economic impact) in the population (1,2,3,4). Since the vaccine is reformulated each season according to the virus serotypes in circulation, its effectiveness can vary from season to season. Vaccine effectiveness is defined as the relative risk reduction in vaccinated individuals in observational studies. In addition, other factors such as age and co-morbidities can influence influenza vaccine effectiveness (IVE). Thus, the seasonal IVE should be monitored annually (1,5,6,7).

In Turkey, national sentinel influenza surveillance has been carried out since 2005. A total of 180 family physicians in 17 provinces of Turkey participate in this surveillance. Participating physicians collect respiratory specimens from a patient with influenza-like illness (unexplained fever >38 °C and cough and/or sore throat) and send them weekly to 1 of the 3 Turkish national influenza reference laboratories. The specimens are tested for influenza and other pathogens. The choice of patients from which to collect specimens is at the discretion of the treating physician. In Turkey, the weekly influenza incidence in the influenza season is generally over 100 per 100.000 people according to the surveillance system (8). Trivalent seasonal influenza vaccines are recommended for routine use and reimbursed for the following people: healthcare workers, ≥65 years of age, living in nursing homes, have underlying co-morbidities (e.g. chronic pulmonary diseases including asthma, chronic cardiac diseases, any chronic metabolic diseases, chronic renal dysfunction, hemoglobinopathies, immunodeficiency, or receiving immunosuppressive treatment), 6 months to 18 years of age and on long-term acetylsalicylic acid treatment. Influenza vaccines are also available at cost to others who do not meet these criteria (8).

Monitoring IVE in a population can help determine community vaccination policies. However, IVE in Turkey has never been determined before. To measure IVE, observational studies are used, including the test-negative case-control study design, where laboratory-confirmed cases of influenza are compared to test-negative controls. This study type is used to estimate IVE in many countries, but has not been previously used in Turkey (9,10,11,12,13,14,15,16). In the 2014-2015 influenza season, we used this design for the first time in Turkey, and we aimed to estimate IVE in preventing laboratory-confirmed influenza with data collected from the national sentinel surveillance system. Such a study conducted with surveillance data can also provide information that can serve to improve influenza surveillance.

## MATERIALS AND METHODS

### Study period and population

Influenza surveillance data was used to estimate IVE in this study. The study population consisted of medically attended individuals with influenza-like illness whose respiratory specimens were sent for testing in the 2014-2015 influenza season. Influenza season was defined as the period that started the week in which the first laboratory-confirmed influenza was detected and ended the week of the last detection between 1 August 2014 and 31 July 2015. No population sampling strategy was used.

We used a test-negative case-control design in which vaccination status was compared between cases and controls among individuals who were tested for influenza. Specimens were tested for influenza using real-time reverse transcription polymerase chain reaction (PCR). Cases were defined as individuals whose specimens tested positive for influenza and controls were defined as individuals whose specimens tested negative for influenza. If 2 or more specimens of the same individual were collected within 14 days, the first test-positive sample was taken into account. If all specimens of the same individual collected within 14 days tested negative for influenza, then the first sample was taken into account for analysis. The following individuals were excluded from analysis: age younger than 6 months (not eligible for vaccination), test results or influenza type unknown, controls with missing symptom onset date, or symptom onset date that was more than 7 days before the date of specimen collection.

To allow time for vaccine-induced immune response, individuals who received the influenza vaccine at least 14 days before the onset of their influenza-like symptoms were considered vaccinated. In addition, individuals were considered vaccinated if they reported having received the influenza vaccine, but the date of vaccination was missing (i.e. assumed receipt of vaccine at least 14 days before onset of symptoms). All others were considered unvaccinated.

### Data collection

Influenza test results were obtained from all 3 reference laboratories. Data on gender, age, risk group, date of onset of symptoms, date of specimen collection, specimen type (nasal, nasopharyngeal, throat, nasal plus throat, nasopharyngeal plus throat and nasal plus nasopharyngeal) and influenza vaccination status for the 2014-2015 season with vaccination dates were collected from the forms that physicians filled out. Risk groups for severe influenza were people of age 65 or older, people with underlying clinical conditions (including heart, pulmonary, renal, metabolic and haemotologic diseases, cancer and immunocompromising conditions), pregnant women and patients whose body mass index was >30.

### Statistical methods

IVE was calculated for influenza A or B and separately for influenza A serotypes H1N1, H3N2 and influenza B. To estimate vaccine effectiveness the following formula was used: (1-OR) x 100. The OR in this formula is calculated by dividing the vaccination rate in the case group by the vaccination rate in the control group. It refers to the risk of illness in the vaccinated group compared to the unvaccinated group. The formula gives the percentage of risk reduction in the vaccinated group relative to the unvaccinated group. This percentage is the vaccine effectiveness (17).

IVE adjusted for gender, age groups (6 months to <18 years, 18-64 years, and ≥65 years), risk group (yes/no) and risk period (October 2014-January 2015 vs February 2015-June 2015) was calculated using logistic regression analysis. The dependent variable was vaccination status (0: unvaccinated, 1: vaccinated) and the reference categories for the independent variables were gender: female; age groups: 6 months to <18 years; risk group: no; and risk period: October 2014-January 2015. Logistic regression was performed using the enter method for the inclusion of the independent variables in the models.

The chi-square test was used for comparisons between cases and controls. Confidence intervals around the IVE estimates were also calculated. Sensitivity analysis was performed to understand the effect of the exclusion criteria on the results. For this, the adjusted IVEs were calculated against each type of influenza virus using only 2 exclusion criteria: unknown vaccination status and unknown influenza test/type result. Statistical analyses were performed using Statistical 25 Package for Social Sciences (SPSS) version 15.0 (SPSS Inc., Chicago, IL, USA).

### Ethical approval and informed consent

Ethical approval for the study was provided by Dokuz Eylül University Committee (2015/01-19) and permission for data collection was granted by the Public Health Institute of Turkey. Informed consent was not taken from the individuals.

## RESULTS

**Figure 1:**
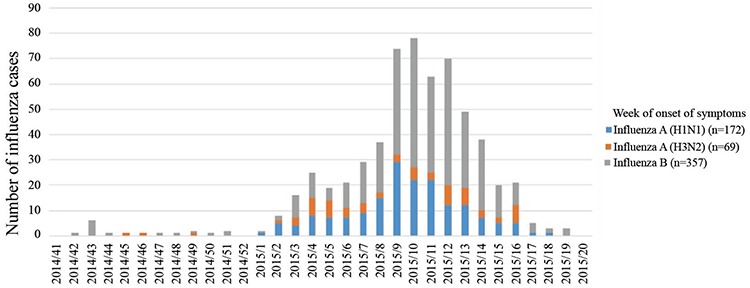
Number of influenza cases by influenza types and week of onset of symptoms in the national sentinel surveillance in Turkey in 2014-2015 influenza season (n=598).

**Figure 2:**
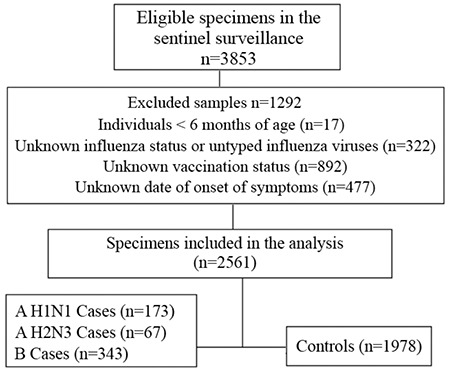
The number of eligible specimens and influenza A (H1N1), A (H3N2) and B cases and controls included in the influenza vaccine effectiveness analysis in 2014-2015 season in Turkey.

During the study period (between 13 October 2014 and 7 June 2015), eligible specimens from 3853 individuals were tested for influenza. Of these, 598 were confirmed as influenza by RT-PCR. Cases by influenza type and week of onset of symptoms are shown in Figure 1. After applying the exclusion criteria, 583 samples tested positive and were eligible for analysis out of 2561 individuals. Hence, there were 583 cases and 1978 controls (those tested negative for influenza). Unknown vaccination status was the major reason (60.0%) for exclusion from analysis (an individual could meet multiple exclusion criteria) (Figure 2).

Of all specimens collected from cases and controls (n=2561), the most frequent specimen type was throat specimens at 42.1% (n=1079). While nasal specimens were collected from 35.7% (n=914) of the study population, both nasal and throat specimens were collected from 18.0% (n=461). All other specimen types (nasopharyngeal specimen, nasopharyngeal plus throat specimen and nasal plus nasopharyngeal specimen) were collected from 4.2% (n=107) of the study population.

Any influenza positivity rate was 22.7% (583/2561). Among positive influenza test results, influenza B (58.8%) was the most frequent type identified, followed by influenza A (H1N1) (29.7%) and A (H3N2) (11.5%).

The vaccination rate was 4.2% (95% CI: 3.5 to 5.0) in the study population. Among all the specimens included in the analysis, the laboratory-confirmed influenza rate was higher in the period from February 2015 to June 2015 (35.2%) than that in the period from October 2014 to January 2015 (6.5%) (p<0.001). When comparing controls with influenza cases, a higher proportion of controls were vaccinated (4.7%) than cases (2.6%) (p=0.025). Characteristics of influenza cases (by influenza type) and controls are shown in Table 1.

Crude IVE against influenza A or B was 46.5% (95% CI: 6.9 to 69.2) and adjusted IVE against influenza A or B was 50.6% (95% CI: 11.5 to 72.4). Adjusted IVE against influenza A (H1N1), A (H3N2) and B was 68.4% (95% CI: -2.9 to 90.3), 75.0% (95% CI: -86.1 to 96.7) and 44.6% (95% CI: -27.9 to 66.6), respectively (Table 2).

### Sensitivity analysis

Using only 2 exclusion criteria (unknown vaccination and unknown influenza status/type), the adjusted IVE estimates against influenza A or B, A (H1N1), A (H3N2) and B were 52.0% (95% CI: -15.7 to 72.6), 61.0% (95% CI: -9.4 to 86.1), 77.2% (95% CI: -69.8 to 96.9), and 41.0% (95% CI: -14.8 to 69.6), respectively.

## DISCUSSION

This is the first study in Turkey where the IVE in preventing laboratory-confirmed influenza was estimated. In this study conducted using the national sentinel surveillance data during the 2014-2015 influenza season in Turkey, IVE in preventing laboratory-confirmed influenza A or B was moderate (50.6%, 95% CI: 11.5 to 72.4). The results also suggest a relatively higher 2014-2015 IVE against influenza A (H3N2) and a moderate 2014-2015 IVE against influenza B and A (H1N1) in preventing laboratory-confirmed influenza. Despite the fairly large sample size of the study, the IVE estimates for the 3 types of influenza viruses were not precise due to the low number of vaccinated individuals especially in influenza cases.

The prcentage of influenza types among all of the positive results was 58.8%, 29.7%, and 11.5% for influenza B, influenza A (H1N1) and A (H3N2), respectively. These data are similar to those reported by the national influenza surveillance system conducted by the Turkish Ministry of Health (8). In a multicentre study involving 8 European countries (I-MOVE multicentre case-control study), the most frequent influenza type among the 5509 influenza cases reported during the 2014-2015 influenza season was influenza A (H3N2) followed by influenza B and influenza A (H1N1) (17). The I-MOVE ranking was not similar to this study and the Turkish influenza surveillance report (8,17). The I-MOVE multicentre case-control study suggested a low 2014-2015 IVE against influenza A (H3N2) (14.4%, 95% CI: -6.3 to 31.0) and a moderate IVE against influenza A (H1N1) (54.2%, 95% CI: 31.2 to 69.9) and B (IVE: 48.0%, 95% CI: 28.9 to 61.9) with more precise estimates than those in our study. The vaccination coverage in the I-MOVE multicentre study was approximately 12%, which was almost three-fold higher than the vaccination coverage in this study (18). According to the end of 2014-2015 influenza season results in the United Kingdom, the IVE for the prevention of laboratory-confirmed influenza B in primary care settings was similar (IVE: 46.3%, 95% CI: 13.9 to 66.5) to our estimate, but the IVE estimation against influenza A (H3N2) was 29.3% (95% CI: -29.9 to 67.5), which was numerically lower than our estimate (19). In those studies that estimated a low IVE against influenza A (H3N2) in the 2014-2015 season, drifted influenza A (H3N2) viruses were documented (18,19). It is possible that drifted influenza viruses might have circulated in the Turkish population during the study period, and we cannot exclude the possibility of a mismatch between the influenza subtypes included in that season’s vaccine and the circulating subtypes.

The influenza vaccination coverage was estimated to be very low (4.2%) in our study. Two influenza vaccination coverage studies conducted in 2006 in Turkey estimated that annual influenza vaccination coverage was less than 15% in the risk groups and 7.4% among the entire study population. However, neither of these studies were population-based, nor representative of the country as a whole (20,21). Therefore, lower vaccination coverage in our study was not surprising, and it suggests that influenza vaccination coverage in Turkey has not been increasing since 2006.

The estimated number of people in a risk group for influenza in Turkey is between 27 and 33 million (22). This group constitutes approximately more than 40% of the entire Turkish population. In our IVE analysis, the proportion of individuals in a risk group was approximately 10%. People who are in a risk group because of a chronic condition present directly to the physician managing their long-term condition, rather than going to their family practitioner and will not have specimens submitted via the sentinel surveillance system. If we assume that more people in the risk group had been vaccinated, influenza viruses in circulation in the Turkish population could be decreased and influenza vaccination impact could be higher in Turkey (23,24,25). In 2 face-to-face surveys conducted in the Istanbul province in Turkey, the first leading factor adversely influencing vaccination among people who said ‘I never get vaccinated’ was disbelief that influenza vaccines are effective (20). Therefore, it is important to document annual IVE in Turkey to better inform the public and influenza vaccination policy makers.

The following are the few limitations of the IVE assessment in this study. In total, 66.4% of eligible specimens could be included in the IVE analysis. However, vaccination rate was not different between the excluded and included groups. The proportion of individuals with unknown vaccination status was 23.1% of total eligible specimens, making it difficult to estimate the potential impact of the unknowns on the IVE.

The test-negative design is less susceptible to bias from misclassification of outcomes (influenza) and is less susceptible to confounding based on health care seeking behaviour. However, as with any other observational study, it is still susceptible to other types of bias and confounding.

Although we adjusted the IVE estimates for gender, age group, risk group and period, there may be residual confounding since the period variable has only 2 categories instead of 4 categories. Since there were no available influenza vaccination records, information on vaccination status was obtained from the form recorded by physicians when specimens of patients were collected. These forms had been filled out based on the patients’ declaration, and therefore the measurement of vaccination status may have been subject to recall bias. In addition, we assumed that vaccinated individuals with missing vaccination dates had received the vaccine at least 14 days before the onset of symptoms; it is unlikely that this led to misclassification, as such individuals would be more likely to remember the date if they were vaccinated within the past 2 weeks. Additionally, as samples were sent at the discretion of the sentinel physician without a defined sampling strategy, it is possible that physicians were more (or less) likely to swab patients known not to have been vaccinated, i.e. testing is applied differentially and varies with the likelihood of immunisation or exposure.

The IVE estimates had low precision due to the small sample size and the small number of vaccinated individuals [especially in influenza A (H3N2) cases]. The IVE estimates for influenza A (H1N1), A (H3N2), and B did not reach statistical significance; however, the IVE against influenza A or B overall was statistically significant. Subgroup analysis for age-specific IVE (i.e. for children and those >65 years) is an important consideration, but that stratification by age would have further decreased the statistical power and precision of the estimates, given that the predominant contribution was from adults 18 to <65 years. Results of the sensitivity analysis assessing the impact of exclusion criteria on IVE showed that the recalculated adjusted IVE estimates were similar to the adjusted IVE estimates in the original analysis. This indicates that exclusions based on other criteria (unknown date of onset of symptoms and specimens collected more than 7 days after onset of symptoms) did not impact IVE.

In summary, we calculated the IVE in preventing laboratory-confirmed influenza in the Turkish population for the first time using national sentinel surveillance data in 2014-2015 season. The overall IVEs against influenza A or B were moderate. The IVE against influenza A (H1N1) and B was also moderate with low precision, but the IVE point estimate against influenza A (H3N2) was higher than those in other European studies. The study population had a low vaccination rate, showing that influenza vaccination coverage in Turkey needs improvement to control influenza morbidity and mortality. The coverage estimate should be confirmed in the risk groups in particular by further studies specifically designed to estimate vaccination coverage. In addition, further studies evaluating IVE for the prevention of influenza hospitalisations and deaths in Turkey are necessary. Annual IVE in Turkey should continue to be monitored as part of the national sentinel influenza surveillance and the surveillance should be strengthened to estimate IVE with more precision. National surveillance systems are important in monitoring influenza circulation and IVE estimation and supporting international efforts to prevent influenza across borders.

## Figures and Tables

**Table 1:**
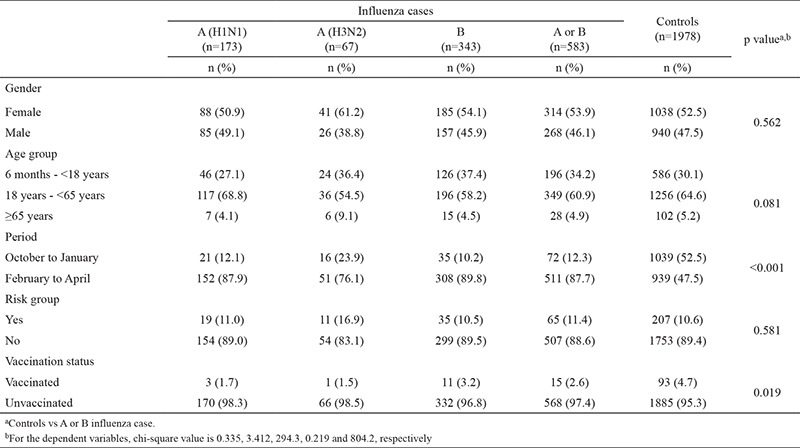
Demographic characteristics and period of occurence of influenza A (H1N1), A (H3N2) and B cases and controls in the analysis in 2014-2015 season in Turkey (n=2561)

**Table 2:**
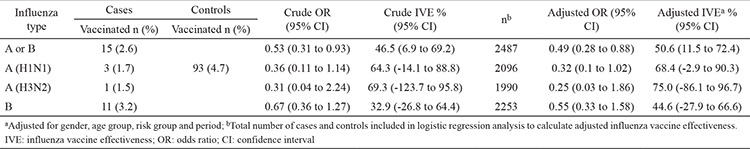
Crude and adjusted influenza vaccine effectiveness estimates against influenza A (H1N1), A (H3N2) and B in 2014/2015 season in Turkey

## References

[1] No authors listed (2012). Vaccines against influenza WHO position paper - November 2012. Wkly Epidemiol Rec.

[2] Glezen WP (1982). Serious morbidity and mortality associated with influenza epidemics. Epidemiol Rev.

[3] Thompson WW, Shay DK, Weintraub E, Brammer L, Bridges CB, Cox NJ, et al (2004). Influenza-associated hospitalizations in the United States. JAMA.

[4] Nichol KL, Nordin JD, Nelson DB, Mullooly JP, Hak E (2007). Effectiveness of influenza vaccine in the community-dwelling elderly. N Engl J Med.

[5] Manzoli L, Ioannidis JP, Flacco ME, De C, Villari P (2012). Effectiveness and harms of seasonal and pandemic influenza vaccines in children, adults and elderly: a critical review and re-analysis of 15 meta-analyses. Hum Vaccin Immunother.

[6] Osterholm MT, Kelley NS, Sommer A, Belongia EA (2012). Efficacy and effectiveness of influenza vaccines: a systematic review and meta-analysis. Lancet Infect Dis.

[7] Kelly H, Steffens I (2013). Complexities in assessing the effectiveness of inactivated influenza vaccines. Euro Surveill.

[8] (2015). Turkish Ministry of Health, Public Health Institution, Department of Infectıous Diseases. Weekly influenza surveillance report.

[9] Orenstein WA, Bernier RH, Hinman AR (1988). Assessing vaccine efficacy in the field. Further observations. Epidemiol Rev.

[10] Valenciano M, Kissling E, Ciancio BC, Moren A (2010). Study designs for timely estimation of influenza vaccine effectiveness using European sentinel practitioners networks. Vaccine.

[11] Jefferson T, Rivetti D, Rivetti A, Rudin M, Di C, Demicheli V (2005). Efficacy and effectiveness of influenza vaccines in elderly people: a systematic review. Lancet.

[12] Castilla J, Martínez-Artola V, Salcedo E, Martínez-Baz I, Cenoz MG, Guevara M, et al (2012). Vaccine effectiveness in preventing influenza hospitalizations in Navarre, Spain, 2010-2011: cohort and case-control study. Vaccine.

[13] Kissling E, Valenciano M, Cohen JM, Oroszi B, Barret AS, Rizzo C, et al (2011). I-MOVE multi-centre case control study 2010-11: overall and stratified estimates of influenza vaccine effectiveness in Europe. PLoS One.

[14] Pebody R, Hardelid P, Fleming D, McMenamin J, Andrews N, Robertson C, et al (2011). Effectiveness of seasonal 2010/11 and pandemic influenza A(H1N1)2009 vaccines in preventing influenza infection in the United Kingdom: mid-season analysis 2010/11. Euro Surveill.

[15] Puig-Barberà J, Arnedo-Pena A, Pardo-Serrano F, Tirado-Balaguer MD, Pérez-Vilar S, Silvestre-Silvestre E, et al (2010). Effectiveness of seasonal 2008-2009, 2009-2010 and pandemic vaccines, to prevent influenza hospitalizations during the autumn 2009 influenza pandemic wave in Castellón, Spain. A test-negative, hospital-based, case-control study. Vaccine.

[16] De G, Skowronski DM, Wu XW, Ambrose CS (2013). The test-negative design: validity, accuracy and precision of vaccine efficacy estimates compared to the gold standard of randomised placebo-controlled clinical trials. Euro Surveill.

[17] Weinberg GA, Szilagyi PG (2010). Vaccine epidemiology: efficacy, effectiveness, and the translational research roadmap. J Infect Dis.

[18] Valenciano M, Kissling E, Reuss A, Rizzo C, Gherasim A, Horváth JK, et al (2016). Vaccine effectiveness in preventing laboratory-confirmed influenza in primary care patients in a season of co-circulation of influenza A(H1N1)pdm09, B and drifted A(H3N2), I-MOVE Multicentre Case-Control Study, Europe 2014/15. Euro Surveill.

[19] Pebody R, Warburton F, Andrews N, Ellis J, von B, Robertson C, et al (2015). Effectiveness of seasonal influenza vaccine in preventing laboratory-confirmed influenza in primary care in the United Kingdom: 2014/15 end of season results. Euro Surveill.

[20] Biberoglu K (2006). Haydi büyükler aşıya. Actual Med.

[21] Oncel S, Turhan O, Huseyin PH, Yalcin AN (2008). Status of influenza vaccination in patients presenting to two neighborhood primary health care clinics in Antalya. Infez Med.

[22] Ciblak MA;, Grip Platformu (2013). Influenza vaccination in Turkey: prevalence of risk groups, current vaccination status, factors influencing vaccine uptake and steps taken to increase vaccination rate. Vaccine.

[23] Torvaldsen S, McIntyre PB (2002). Observational methods in epidemiologic assessment of vaccine effectiveness. Commun Dis Intell Q Rep.

[24] Hanquet G, Valenciano M, Simondon F, Moren A (2013). Vaccine effects and impact of vaccination programmes in post-licensure studies. Vaccine.

[25] Halloran ME, Struchiner CJ, Longini Jr (1997). Study designs for evaluating different efficacy and effectiveness aspects of vaccines. Am J Epidemiol.

